# Manubriosternal dislocation type II associated with thoracic spine fracture: What can we learn from this?

**DOI:** 10.7196/AJTCCM.2019.v25i3.005

**Published:** 2019-09-17

**Authors:** N N M Razafimanjato, T D N Ravelomihary, N O N L H Rajaonarison, A Ahmad, H J L Rakotovao

**Affiliations:** 1 Thoracic Surgery Unit, Joseph Ravoahangy Andrianavalona Hospital and Faculty of Medicine, University of Antananarivo, Madagascar; 2 Imaging Medical Centre, Joseph Ravoahangy Andrianavalona Hospital and Faculty of Medicine, University of Antananarivo, Madagascar

**Keywords:** chest trauma, manubriosternal joint, sternum, spine, sternal segment dislocation, thoracic surgery

## Abstract

Traumatic dislocation of the manubriosternal joint is rare, and management correction requires an individualised decision. We report a case of a young female who suffered a type II manubriosternal dislocation with concomitant thoracic spine fracture, as a result of a motor vehicle accident. The mechanism and various therapeutic outcomes are discussed, and a review of the literature provided.

## Background

Type II manubriosternal dislocations with concomitant spinal fracture are extremely rare, and may be associated with thoracic visceral injuries. There are very few published case reports.^[1–3]^ There is no definite standard for treating such injuries, and in practice, the thoracic surgeon often has difficulty choosing and planning surgical technique(s). We describe a case of a young woman with traumatic manubriosternal dislocation. In this literature review, the main pathophysiological mechanism of traumatic manubriosternal dislocation and spine fracture, and their treatments, are discussed.

## Case report

**Fig. 1:**
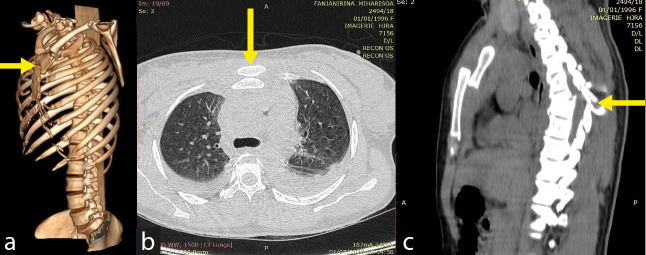
(a) Left sagittal view of 3D CT scan showing manubriosternal joint dislocation type II: anterior dislocation of the sternal body; (b) Axial CT scan showing manubriosternal joint dislocation and bilateral haemothorax; (c) Sagittal CT image showing three column injuries: fracture of body of dorsal vertebrae T5, T6, T7. CT = computed tomography

A 22-year-old woman was admitted to our emergency department as a result of a motor-vehicle accident. At presentation, the patient had stable vital signs. She was semi-conscious with a Glasgow Coma Score (GCS) of 15, and a palpable deformity at the manubriosternal joint that was tender to the touch. On examination, the patient had stable vital signs. Neurological examination revealed a partial neurological deficit. Multiple ecchymoses were found all over her body. The rest of the examination was unremarkable. Oxygen saturation was 92% with face-mask oxygen. A left thoracostomy drain was inserted on a clinical basis. She was managed in the intensive care unit (ICU). A computed tomography scan of the chest (CT chest) and neck revealed a manubriosternal dislocation fracture, bilateral haemothorax with lung contusions and fractures of dorsal vertebrae T5, T6 and T7 (Fig. 1A-C). After initial management at the emergency department, she was kept in the ICU. Even with well-managed treatment, the patient remained in persistent neurogenic shock due to acute spinal cord injury, and had severe hypotension and bradycardia from thoracic spine trauma. On day 3 of admission she developed cardiovascular dysfunction that was unresponsive to any therapeutic manoeuvres. The patient did not respond to resuscitation measures and subsequently died.

## Discussion

### Epidemiology

Sternal dislocation may present as manubriosternal joint dislocation (MSJD) or sternal segment dislocation.^[4]^ Manubriosternal dislocations are a rare occurrence, representing 17.6% of lesions of the sternum, corresponding to <0.5% of all traumatic lesions.^[1-3,5]^ Of 250 fatal road accidents, this type of lesion was observed in 13 patients, and was associated with fracture of the thoracic spine in just 3 individuals.^[2]^ Fowler *et al*. ^[6]^ reported 21 patients with dislocation fractures of the sternum, in 9 of whom fractures of the thoracic vertebrae were diagnosed. In another analysis of 1 124 motor-vehicle collision victims over a 3-year period, an increase of sternal injury from 0.7% to 4% occurred, and the increase was mainly associated with the introduction of seat belts.^[7]^

### Pathophysiology and classification

Sternal injuries are frequently caused by falls from heights, or indirect trauma due to spinal flexion-compression injury. The most common mechanisms accounting for sternal fractures are motor vehicle collisions and blunt trauma to the chest and abdomen.^[8]^ Some morphological characteristics, such as kyphoscoliosis of the thoracic spine and joint alterations in patients with rheumatoid arthritis, predispose patients to manubriosternal luxation.^[2,9,10]^ Thirupathi and Husted^[11]^ distinguished two types of manubriosternal dislocations: the sternal body may be dislocated either posteriorly (type I) or anteriorly (type II) to the manubrium. Direct injury to the chest is the mechanism usually involved in type I (usually seen with direct impact or compression injury to the anterior chest),^[3]^ whereas deceleration injuries resulting in hyperflexion with compression injury to the upper thorax, rheumatoid disease and kyphosis are the predisposing factors for type II. It is usually associated with flexion-compression upper thoracic spine fractures, and requires tremendous force.^[4,12]^ The most common associated injuries are rib fractures, pulmonary contusion, pneumothorax and extremity fractures. One study showed an 18% incidence of myocardial contusion associated with sternal fractures. ^[12]^ Type II injuries may be associated with injuries to the aorta, other great vessels, the trachea and the oesophagus, which are potentially lifethreatening.^[13]^ In our patient, the manubrium was posteriorly displaced, categorising it as type II, and the mechanism was flexion-compression caused by indirect trauma.

### Diagnosis

Clinical examination of the patient is essential in the diagnosis of manubriosternal luxation/dislocation, and can reveal deformity and pain in the region of the manubriosternal joint. Gopalakrishnan *et al*. ^[14]^ state that in the absence of any clinical signs, mediastinal widening is due to paravertebral haematoma in the absence of aortic injury. In our case, the contrast CT chest did not show any mediastinal or vascular injury. Micro-CT is an effective tool for the diagnostic evaluation and therapeutic management of sternum anatomy. In addition to diagnosis, it is necessary to measure the length of the screws in osteosynthesis, and to calculate whether the sternum will be amenable to effective plate and screw fixation. Screw lengths should be no greater than 21 mm, and no less than 5.2 mm.^[15]^

### Indications for therapeutics

A systematic review of the literature published from 1990 to June 2017 concluded that treatment of traumatic sternal fractures and dislocations is an underexplored topic, because limited research has been performed on indicated treatment outcomes.^[5]^

The first case of surgical correction of the sternal fracture was described by McKim^[16]^ in 1943. Surgical stabilisation of traumatic sternal fractures and dislocations without associated lesions remains controversial, and requires an individualised decision. Most (>95%) sternal injuries are treated conservatively. In general, non-operative treatment is preferred for undisplaced fractures or dislocations. Woo^[17]^ reported on the conservative treatment of manubriosternal dislocation, consisting of manipulative hyperflexion reduction and rest, but no prognosis for this method of treatment was provided.^[18]^ Similarly, Pidcoe *et al*. ^[18]^ suggested rehabilitation of type II manubriosternal dislocation consisting of progressive compressive and tensile loads placed on the non-union, which appears to stimulate the recovery process.^[18]^ This technique is called ‘distraction osteogenesis’, and is indicated favourably for the treatment of pseudarthrosis or malunion of dislocation.^[19]^ However, as reported by Harston and Roberts,^[20]^ surgical fixation is indicated in cases of displaced fractures, persistent pain affecting respiration, chronic non-union and sternal instability.^[3,4]^ Operative treatment is also indicated to avoid a complication such as manubriosternal septic arthritis.^[10]^

In the paediatric population, Murala and Nunn^[21]^ recommend operative immobilisation of the joint (not necessarily anatomically perfect reduction) through a small incision, in symptomatic children with traumatic manubriosternal dislocation, to relieve pain and prevent further deformity. The joint remodels itself in a growing child.

Various operative procedures using plates, wire loops, Kirschner wires or polydioxanone loops have been described in the literature.^[1,22]^ Schmitz *et al*. ^[23]^ report a resection arthroplasty for luxation of the manubriosternal joint in rheumatoid arthritis, with success. The excision of a manubriosternal synchondrosis already damaged by a traumatic event, and the cuneiform chondrectomy of the third and fourth rib cartilage bilaterally, was the technique used by Divisi *et al*.,^[24]^ with an acceptable outcome. Use of demineralised bone matrix can facilitate bone callus formation, owing to the osteo-inductive activity and the compatibility of the surrounding blood cells.^[22]^ Klei *et al*. ^[5]^ undertook a systematic review of current treatment and outcomes of traumatic sternal fractures, and concluded that both surgical and conservative treatment of traumatic sternal fractures and dislocations seem to be safe and effective. All patients evaluated in this review displayed sternal healing, while reported complication rates were as low as 3%.

Manubriosternal injury represents a serious injury, due to commonly associated potentially life-threatening injuries. Some authors have reported mortality rates in patients with sternal fractures ranging from 24% to 45%.^[8]^ This high mortality rate is due to associated thoracic, pulmonary, cardiac and spinal injuries.^[8]^

## Conclusion

The paucity of literature on Type II MSJD with concomitant spinal fracture has delayed a consensus on its surgical management. We conclude that a CT chest with contrast is indicated to rule out visceral injuries, and to plan intra-operative surgical technique. We recommend that operative treatment is indicated to avoid complications associated with manubrosternal dislocation.
